# Heat shock factor 1 inhibition enhances the effects of modulated electro hyperthermia in a triple negative breast cancer mouse model

**DOI:** 10.1038/s41598-024-57659-x

**Published:** 2024-04-08

**Authors:** Pedro H. L. Viana, Csaba A. Schvarcz, Lea O. Danics, Balázs Besztercei, Kenan Aloss, Syeda M. Z. Bokhari, Nino Giunashvili, Dániel Bócsi, Zoltán Koós, Zoltán Benyó, Péter Hamar

**Affiliations:** 1 Institute of Translational Medicine, Semmelweis University, Tűzoltó Utca 37-47, Budapest, 1094 Hungary; 2 HUN-REN-SU Cerebrovascular and Neurocognitive Diseases Research Group, Tűzoltó Utca 37-47, Budapest, 1094 Hungary

**Keywords:** Triple-negative breast cancer, Modulated electro-hyperthermia, Heat shock response, HSF1, Hsp70, Cancer, Diseases, Oncology

## Abstract

Female breast cancer is the most diagnosed cancer worldwide. Triple negative breast cancer (TNBC) is the most aggressive type and there is no existing endocrine or targeted therapy. Modulated electro-hyperthermia (mEHT) is a non-invasive complementary cancer therapy using an electromagnetic field generated by amplitude modulated 13.56 MHz frequency that induces tumor cell destruction. However, we have demonstrated a strong induction of the heat shock response (HSR) by mEHT, which can result in thermotolerance. We hypothesized that inhibition of the heat shock factor 1 (HSF1) can synergize with mEHT and enhance tumor cell-killing. Thus, we either knocked down the HSF1 gene with a CRISPR/Cas9 lentiviral construct or inhibited HSF1 with a specific small molecule inhibitor: KRIBB11 in vivo. Wild type or HSF1-knockdown 4T1 TNBC cells were inoculated into the mammary gland’s fat pad of BALB/c mice. Four mEHT treatments were performed every second day and the tumor growth was followed by ultrasound and caliper. KRIBB11 was administrated intraperitoneally at 50 mg/kg daily for 8 days. HSF1 and Hsp70 expression were assessed. HSF1 knockdown sensitized transduced cancer cells to mEHT and reduced tumor growth. HSF1 mRNA expression was significantly reduced in the KO group when compared to the empty vector group, and consequently mEHT-induced Hsp70 mRNA upregulation diminished in the KO group. Immunohistochemistry (IHC) confirmed the inhibition of Hsp70 upregulation in mEHT HSF1-KO group. Demonstrating the translational potential of HSF1 inhibition, combined therapy of mEHT with KRIBB11 significantly reduced tumor mass compared to either monotherapy. Inhibition of Hsp70 upregulation by mEHT was also supported by qPCR and IHC. In conclusion, we suggest that mEHT-therapy combined with HSF1 inhibition can be a possible new strategy of TNBC treatment with great translational potential.

## Introduction

Female breast cancer is the most diagnosed cancer type and has surpassed lung cancer as the leading cause of global cancer incidence in 2020, with an estimated 2.3 million new cases, representing 11.7% of all cancer cases^1^. Triple-negative breast cancer (TNBC) lacks expression of estrogen receptor (ER), progesterone receptor (PR), and human epidermal growth factor receptor 2 (HER2), and is the most aggressive type accounting for 15–20% of all breast cancer cases^2^ with poor prognosis^3^. As TNBC lacks targetable receptor expression, there is no endocrine or targeted therapy available at present. Thus, effective TNBC treatment regimens are still lacking^4^. Therefore, development of new TNBC treatment strategies is an unmet clinical need^5^.

Modulated electro-hyperthermia (mEHT) treatment (Oncothermia) is a non-invasive complementary cancer therapy applied in the clinics successfully^6–10^. mEHT uses an electromagnetic field generated by amplitude modulated 13.56 MHz frequency that induces tumor cell destruction^11^. The modulated radiofrequency (RF) energy is directed to the tumor area and creates a heat flow that triggers biochemical processes in the malignant cell membrane, causing apoptotic cell death^12,13^. The cell killing effects are targeted due to differences between the electromagnetic properties of lipid rafts (electrical conductivity and permeability) of cancer cells and surrounding normal tissue^14^. Unlike convetional hyperthermia the surrounding normal tissues are minimally damaged during mEHT treatments^15^. The specific radiofrequency has been clinically used to treat tumors for approximately 20 years^16^.

The mEHT-induced energy absorbed by tumor tissues results in temperature elevation, hence inducing the heat shock response (HSR)^17^. The HSR, initiated by its master regulator, the heat shock transcription factor 1 (HSF1), protects cells from a wide range of stresses, including heat stress^18^. As described before, cell survival is achieved through the activation of anti-apoptotic proteins and the inhibition of pro-apoptotic proteins, such as Hsp70, a phenomenon known as thermotolerance, which enables cancer cells to withstand the effects of heat^19^. Hsp70 is a molecular chaperone able to restore the balance of cell proteome by protecting proteins and refolding unfolded proteins under a variety of stress conditions^20,21^. Hsp70 is upregulated in various cancer cells promoting survival and proliferation^22^. Therefore, blocking or silencing the HSR by targeting HSF1 or Hsps can be an effective way to reverse thermotolerance in cancer cells.

We previously demonstrated in vitro that inhibition of Hsp70 synergized with mEHT and enhanced its anti-cancer therapeutic effects^17,23^. Moreover, we observed in vivo a strong Hsp70 signal in mEHT-treated tumors around the apoptotic, damaged area^17^. It has been demonstrated that overexpression of Hsp70 in cancer cells is a consequence of the disruption in HSF1 transcriptional activity^24,25^, although Hsp70 might be also expressed regardless of HSF gene^26^. Constitutive activation of HSF1 during tumorigenesis is one possible explanation of the enhanced Hsp70 expression in several cancers^24^; however, the mechanism remains unclear. The inhibition of HSF1 using short hairpin RNAs (shRNAs) or small synthetic molecules, such as KRIBB11, decreased the expression of HSF1 downstream proteins^27^. Furthermore, we also demonstrated in vitro the enhancement of mEHT-4T1 cancer cell killing effect by KRIBB11^23^. KRIBB11 has abolishing activity on the heat shock-dependent induction of Hsp70 gene through inhibition of HSF1, causing significant decrease of tumor growth^28^. Therefore, reducing HSF1 could significantly inhibit the growth of cancer cells and promote their apoptosis^29^. Despite of several HSF1 inhibitors have been investigated^25^, KRIBB11 is the first specific inhibitor of HSF1^28^.

Another way to block the HSR is by gene-silencing its master regulator: HSF1. This strategy has been successfully applied in many studies to demonstrate the pro-oncogenic effects of HSF1^30^. Indeed, it has been demonstrated that HSF1 knockout (KO) mice had decreased susceptibility to spontaneous lymphoma^31^ and survival of tumor bearing mice increased^32^. Furthermore, HSF1-KO prevented tissue hyperplasia and cancer development in a mouse mammary gland-specific HSF1-KO model^33^. An in vitro model using cervical cancer cells has proposed that silencing HSF1 by gene-editing or inhibiting HSF1 by chemical inhibitors sensitizes cancer cells to chemotherapeutic reagents or hyperthermia, in which an incubator was used for heating procedures^34^. Thus, in the present study, we investigated the hypothesis that downregulation of the heat shock response by gene-editing knockdown of HSF1, or by a specific chemical inhibitor of HSF1: KRIBB11, enhances the therapeutic efficiency of mEHT in breast cancer treatment.

## Material and methods

### Cell culture and reagents

For both in vitro and in vivo studies, the 4T1 murine mammary carcinoma cell line, provided by Judy Lieberman (Lieberman Laboratory, Harvard University, Boston, MA, USA), was used. The 4T1 cells were grown as adherent culture in Dulbecco’s Modified Eagle Medium (DMEM high glucose, 4.5 g/L without lglutamine and Phenol Red, Capricorn Scientific, Ebsdorfergrund, Germany, Cat-No. DMEM-HXRXA) supplemented with 10% Fetal Bovine Serum (FBS—South America Origen, EU approved, EuroClone S.p.A., Pero, Italy, CatNo. ECS0180L), l-glutamine 200 mM (Capricorn Scientific, Ebsdorfergrund, Germany, Cat-No. GLN-B), and penicillin/streptomycin 100x (Capricorn Scientific, Ebsdorfergrund, Germany, Cat-No. PS-B). Cells were submitted to passages every 2 or 3 days. Trypsin 10x (Lonza A. G., Basel, Switzerland, Cat-No. 17-160E) was used to release cells from sub-confluent monolayers. The detached cells were seeded back into cell culture flasks or prepared for experiments. KRIBB11 was purchased from MedChemExpress (Monmouth Junction, NJ, USA, Cat-No. HY-100872).

### Construction and verification of stable HSF1-knockdown by flow cytometry

**Figure 1:**
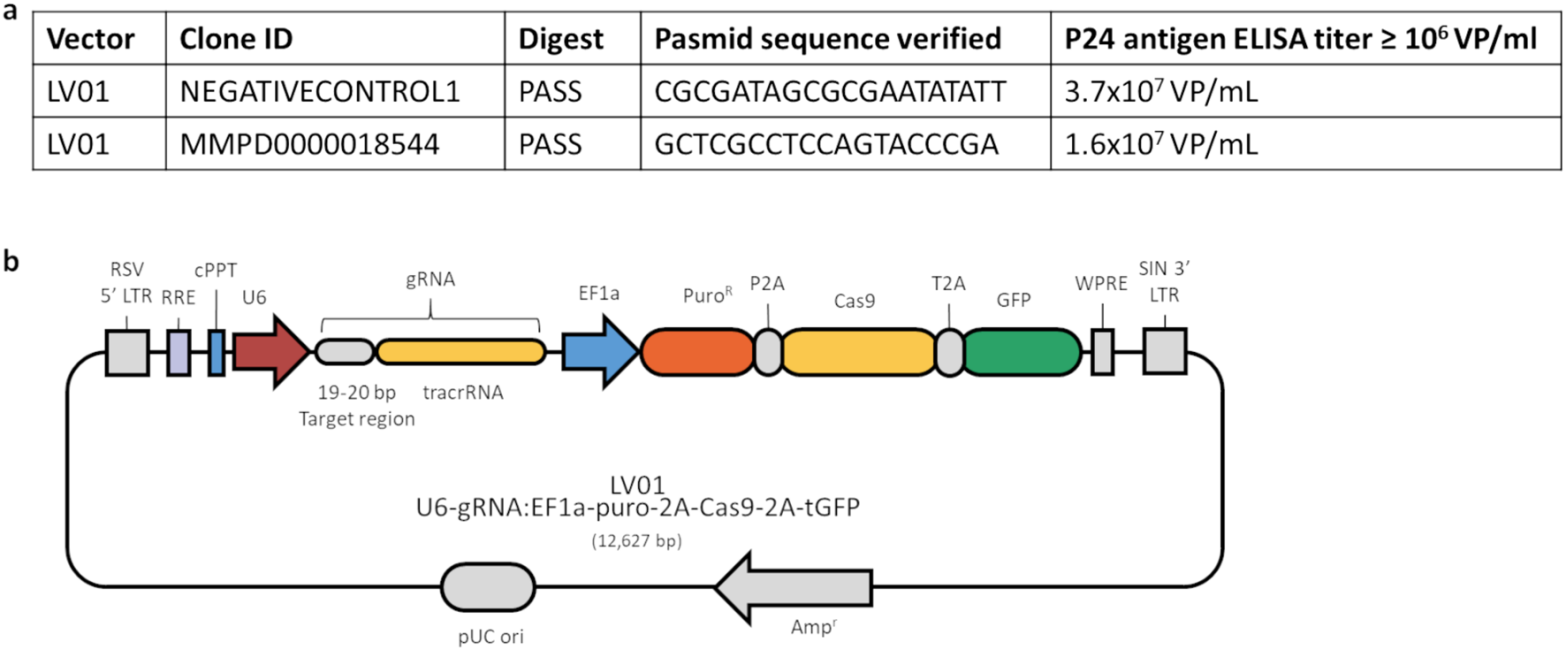
CRISPR/Cas 9 lentiviral constructs. (**a**) Plasmid sequence of the negative control (empty vector) and the HSF1 knockdown, which contains the target gene. (**b**) Scheme showing the structure of HSF1-KO lentiviral particles. (Modified from Sigma-Aldrich^®^ (St. Louis, MO, USA)).

CRISPR gRNA Lentiviral Transduction particles from Sigma-Aldrich^®^ (St. Louis, MO, USA) was used for knockdown of HSF1. The plasmid sequences for HSF1 gene are listed in Fig. 1. The lentiviral structure consisted of (1) the target region to knockdown HSF1-gene; (2) a puromycin resistance gene; and (3) GFP gene. Murine 4T1 cells were seeded at 1.4 × 10^4^ cells/well in triplicate into a 96-well plate and incubated for 24 h at 37 °C. After the cells were adherent to the bottom, 8 µg/mL of hexadimethrine bromide (SigmaAldrich/Merck, St. Louis, MO, USA, Cat-No. H9268-5G) were added to each well to enhance transduction. The lentiviral particles or the negative particles were added to appropriate wells. Cells were further cultured for 24 h, and then the medium containing the lentiviral particles were replaced. Next, transfected cells were selected by culturing in a medium containing 6 µg/ml puromycin (Sigma-Aldrich/Merck, St. Louis, MO, USA, Cat-No. 540411-25MG) to kill non-transfected cells as established earlier^23^. Surviving cells were considered successfully transfected cells. Forty-eight hours after transduction, fluorescence of successful transduced cells was assessed by fluorescent microscope. Puromycin-resistant colonies were selected and expanded to assay for expression of the construct. Cells were sorted by Fluorescence-activated single cell sorting (FACS) (Sony Corp. San Jose, CA, USA, Sony SH800) in three cell types: 4T1 wild type, 4T1 empty vector, and 4T1 HSF1-KO. Flow cytometry was used to check for GFP-positiveness in transduced cells after expansion. For that, cells were harvested at 1 × 10^6^ cells/mL, washed with cold PBS (without Ca & Mg, without Phenol Red, Capricorn Scientific, Ebsdorfergrund, Germany, Cat-No. PBS-1A), and centrifuged for 5 min at 300*g*. The supernatant was removed, and fresh, cold PBS was added. The suspended cells were transferred to FACS tubes and were kept on ice. Cells were analyzed based on GFP-expression by using a CytoFLEX Flow Cytometer (Beckman Coulter, Inc. Brea, CA, USA). The expression of HSF1 and Hsp70 was determined by qPCR.

### In vitro HSF1-KO model

Knockdown-, empty vector-, and wild type 4T1 cells were counted and seeded onto 60 mm petri dishes at a density of 1 × 10^6^ cells per petri dish in DMEM medium with 10% FBS. After 24 h, cells were treated with water bath hyperthermia, 42 °C for 30 min. Two hours after hyperthermia treatment, the medium was discarded and 500 µL TRI Reagent^®^ RT (Molecular Research Center, Inc. Cincinnati, OH, USA, Cat-No. RT 111) was added for RNA collection. The cells were homogenized and transferred to pre-labeled Eppendorf tubes. The Eppendorf tubes were immediately frozen in liquid nitrogen.

### In vivo HSF1-KO model

**Figure 2:**
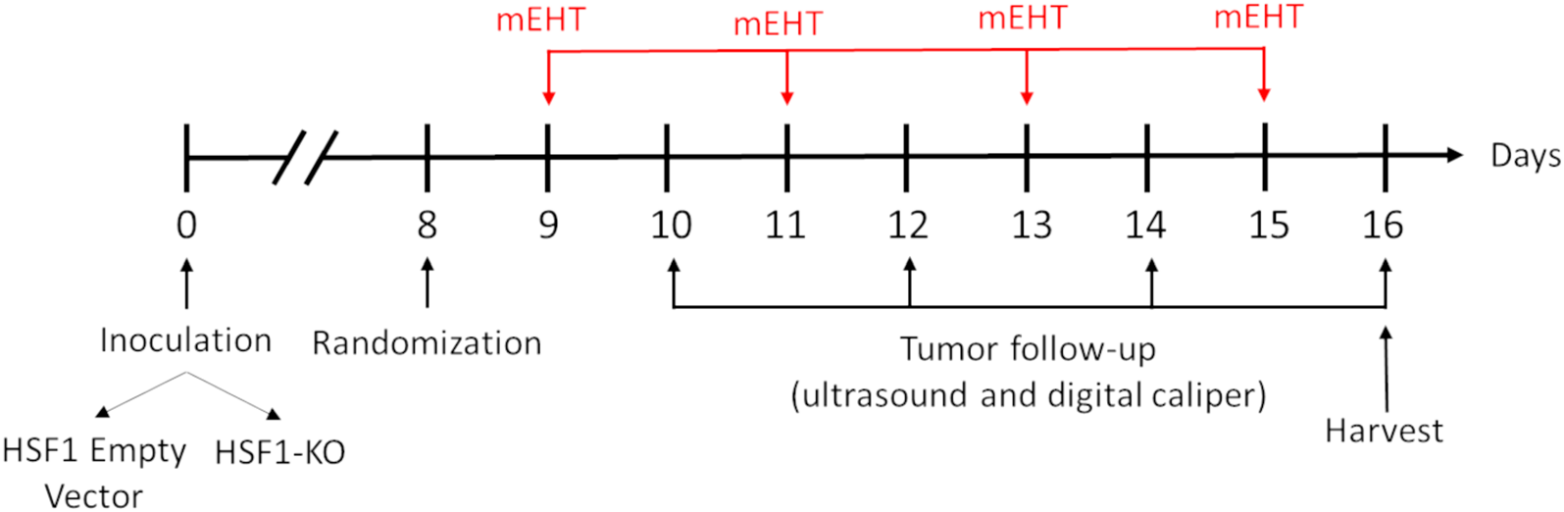
Experimental scheme of the 4T1 HSF1 knockdown model. 4T1 TNBC cells, containing either the HSF1 empty vector (EV) lentiviral construct or the HSF1 knockdown (HSF1-KO) lentiviral construct, were inoculated at day zero. Mice were randomized into groups (Sham EV or mEHT EV, and Sham HSF1-KO or mEHT HSF1-KO) at day 8. mEHT treatments were performed every two days. Tumor volume was monitored by ultrasound and digital caliper between mEHT treatments. The study was terminated on day 16 with the harvest of tumors.

Six-to-eight-weeks old female BALB/c mice were kept under 12 h dark/light cycles with ad libitum access to food and water in the Animal Facility Department of Basic Medical Center, Semmelweis University—Budapest, Hungary. The mice were anesthetized with isoflurane (Baxter International Inc., Deerfield, IL, USA). Anesthesia was induced with 5% concentration and maintained with 2–2.5% concentration in 0.6–0.8 l/min compressed airflow. 4T1 cells were kept in cell culture flasks, trypsinized, counted, suspended in Dulbecco’s PBS 1x (without Ca & Mg, without Phenol Red, Capricorn Scientific, Ebsdorfergrund, Germany, Cat-No. PBS-1A). 1 × 10^6^ 4T1 cells suspended in 50 µL PBS were subcutaneously inoculated into the 4th mammary gland’s fat pad of each mouse^35^ using a 50 µL Hamilton syringe (Hamilton Company, Reno, NV, USA). Eight days after inoculation, tumor size was measured with digital caliper (Fine Science Tools lnc., Foster City, CA, USA) and ultrasound (Phillips Sonos 5500, Philips, Amsterdam, Netherlands) as described earlier^23^. Mice were randomized into treatment groups (Table 1) according to their tumor volume and body mass, to achieve similar average for all groups. Animals exhibiting disproportionate tumor measures, such as irregular shapes, twin tumors, or those where the size significantly deviated from the average, were excluded from the experiment. mEHT treatments were started the day after randomization. In total, four mEHT treatments were performed every two days. In the days between treatments, tumor size was followed by both digital caliper and ultrasound (US). The correlation between tumor mass and US or caliper was colletcted in the Supplementary Fig. S1. Figure 2 shows a schematic overview of HSF1-KO and mEHT treatments schedule. Mice were kept under isoflurane anesthesia for all procedures described here. For tumor sample collection 24 h after the last treatment, heparin 10x (Teva, Debrecen, Hungary, Cat-No. OGYI-T-2216/01) was injected intraperitoneally. Mice physical body condition was checked, and mice were terminated by cervical dislocation. The abdominal cavity was opened, blood was taken, and tumors were harvested, cleaned (from adjacent connective tissues), weighed, and halved in two similar halves: one half was placed in 4% formaldehyde solution (Semmelweis Pharmacy, Budapest, Hungary) and sent to histological processing; the other half was frozen in liquid nitrogen for molecular analysis. Interventions and housing of the animals conformed to the Hungarian Laws No. XXVIII/1998 and LXVII/2002 about the protection and welfare of animals, and the directives of the European Union. All animal procedures were approved by the National Scientific Ethical Committee on Animal Experimentation under the No. PE/EA/50-2/2019.

**Table 1:** HSF-KO experiment number of mice per group.

	Empty vector	HSF1-KO
Sham	6	5
mEHT	8	8

### In vivo KRIBB11 model

**Figure 3:**
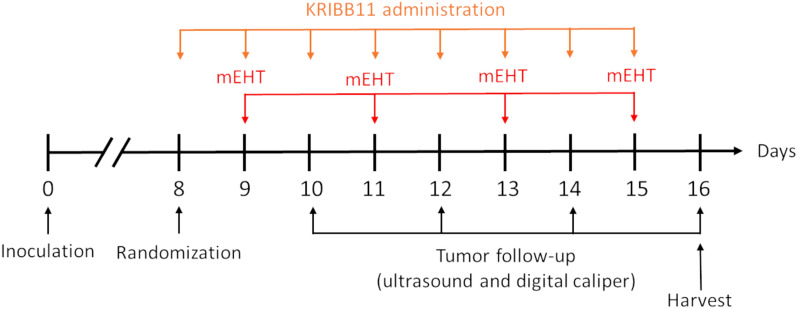
Experimental scheme of the mEHT + KRIBB11 experiment. 4T1 cells were inoculated at day zero. Mice were randomized into groups, as depicted in Table 2, at day 8 together with the first KRIBB11 injection. KRIBB11 was administered every day for 8 days. mEHT treatments were performed every two days. Tumor volume was monitored by ultrasound and digital caliper between mEHT treatments. The study was terminated on day 16 with the harvest of tumors.

KRIBB11 (MedChemExpress, Monmouth Junction, NJ, USA, CatNo. HY-100872) was dissolved in 10% dimethylacetamide (MedChemExpress, New Jersey, USA, Cat-No. HY-W042416), 50% PEG300 (MedChemExpress, Monmouth Junction, NJ, USA, Cat.No.: HY-Y0873), and 40% nuclease-free water (Invitrogen, Carlsbad, CA, USA, Cat-No. 10977-035), as described previously^28^. Following randomization as described above, KRIBB11 or vehicle was administrated intraperitoneally at a dose of 50 mg/kg/day for 8 days according to each group (Table 2). Figure 3 depicts a schematic overview of mEHT + KRIBB11 experiment. Tumor volume was followed as described above. Supplementary Fig. S2 shows the correlation between tumor mass and US (S2a) or caliper (S2b). In total, four mEHT treatments were performed every two days. On day 16, the mice were sacrificed, and the tumors were removed, halved, and analysed by: (1) immunohistochemistry (IHC) and (2) qRT-PCR.

**Table 2:** KRIBB11 experiment number of mice per group.

	Vehicle	KRIBB11
Sham	4	7
mEHT	4	8

### In vivo mEHT treatments

Tumor-bearing mice were treated with modulated electro-hyperthermia (mEHT) device as described in detail earlier^17^. Briefly, electromagnetic heating was generated by capacitive coupled, amplitude modulated 13.56 MHz radiofrequency (LabEHY 200, Oncotherm kft., Budaors, Hungary). The mice were mEHT-treated four times every two days for 30 min plus maximum 5 min for device stabilization with 0.2–1.0 W. Temperature monitoring was performed with optical sensors (Luxtron FOT Lab Kit, LumaSense Technologies, Inc., CA, USA). The optical sensors were calibrated before each treatment and were placed (1) on the skin right above the tumor, (2) into the rectum for body temperature monitoring, (3) on the heating pad, and (4) near the treatment setup for room temperature monitoring. The skin temperature was kept at 40 ± 0.5 °C during the treatments, as it assured the required 42 °C inside the tumor. Rectal temperature was kept in the physiologic range (37.0 ± 0.5 °C), and the heating pad was set at the same temperature. Room temperature was 25 ± 1 °C. For Sham treatments, cables were disconnected, therefore, no electromagnetic field was generated, and no energy was transferred (no heating).

### Histopathology and immunohistochemistry

Formalin-fixed cancer samples were dehydrated and embedded in paraffin. Serial secrions (2.5 µm) were cut for hematoxylin–eosin (H&E) staining or dewaxed and rehydrated for immunohistochemistry (IHC) using a polymerperoxidase system (Histols, Histopathology Ltd., Pécs, Hungary). H&E and stained slides were digitalized using Pannoramic Scan and analyzed with the HistoQuant module of CaseViewer image-analysis software (all from 3DHISTECH, Budapest, Hungary) based on image color and intensity segmentation. The tumor area was digitally annotated and the damaged and living areas were delimited. The ratio between the damaged area per the whole tumor area was used for calculating the tumor destruction ratio (TDR%) on H&E slides.

$$TDR\left( \% \right) = \frac{{\text{Damaged area}}}{{\text{Whole tumor area}}}$$

Slide samples used in IHC were deparaffinized and incubated for 20 min with 3% H_2_O_2_ in methanol to block endogenous peroxidase activity. For antigen retrieval, slide samples were soaked in citrate buffer and heated for 20 min using an Avair electric pressure cooker (ELLA 6 LUX(D6K2A), Bitalon Kft, Pécs, Hungary), followed by 30 min cooling step. 3% bovine serum albumin (BSA, Millipore Corp., Kankakee, IL, USA, CatNo. 82–100-6) solution was used to block non-specific proteins for 20 min. The sections were incubated with the primary antibodies diluted in 1% BSA/TBS + TWEEN (TBST, pH 7.4) (Table 3) overnight in humidity chamber. Peroxidase-conjugated anti-rabbit & anti-mouse IgGs (HISTOLS-MRT, micropolymer-30011.500T, Histopathology Ltd., Pécs, Hungary) were used for 40 min incubation and the enzyme activity was revealed in 3,3′diaminobenzidine (DAB) chromogen/hydrogen peroxide kit (DAB QuantoTA-060-QHDX-Thermo Fischer Scientific, Waltham, MA, USA, Cat-No. 12623957) under optical microscope. All incubations were at room temperature with sample washings between incubations in TBST buffer for 3 × 5 min. Slides were digitalized and the reactions were evaluated. The tumor area was digitally annotated and the area containing positive immune reaction was masked by setting the intensity, color, and saturation in the annotated area on each staining using the QuantCenter module of CaseViewer. The ratio of the masked area to the annotated area (relative mask area = rMA) was used to estimate the expression of the target molecule. rMA of HSF1 and Hsp70 were measured in the intact tumor area. Important to mention, due to technical problems there is no data about Hsp70 staining of three samples from KRIBB11 experiment: one in Sham vehicle, one in Sham KRIBB11, and one in mEHT KRIBB11.

**Table 3:** Antibodies and conditions used for immunohistochemistry.

Antigen	Type	Reference no	Dilution	Vendor1
HSF1	Rabbit, pAb	#4356	1:100	Cell signaling
Hsp70	Rabbit, pAb	#4872	1:100	Cell signaling

### RNA isolation and mRNA RT-PCR

RNA was isolated with the TRI reagent^®^ RT (Molecular Research Center lnc., Cincinnati, OH, USA, Cat-No. RT111) according to the manufacturer’s instructions. RNA integrity was analyzed by agarose gel electrophoresis, sample purity and concentration were measured by a Nanodrop spectrophotometer (Thermo Fisher Scientific Inc., Waltham, MA, USA). Reverse transcription of isolated RNA was performed by High-Capacity cDNA Reverse Transcription Kit (Applied Biosystems Inc., Foster City, CA, USA, Cat-No. 4368814). The amplified cDNA was used as a template for RT-PCR. Gene expression was measured according to standard qPCR procedures with SYBER Green based RT-PCR with SsoAdvanced™ Universal SYBER^®^ Green Supermix (Bio Rad Laboratories, Inc., Hercules, CA, USA, Cat-No. 1725271) on CFX Connect Real-Time PCR Detection System (Bio Rad Laboratories, Inc., Hercules, CA, USA). The relative expression values for the target mRNAs were calculated after normalization using GAPDH. The primers used are listed in Table 4.

**Table 4:** Primers used for RT-PCR.

Gene symbol	Gene name	Primer pairs
GAPDH	Glyceraldehyde-3phosphate-dehydrogenase [*Mus musculus*]	Fwd: CTCCCACTCTTC-CACCTTCG Rev: GCCTCTCTTGCTC-AGTGTCC
Hsp70	Heat shock protein 70 [*Mus musculus*]	Fwd: CTTCACCTCCAA-GTTCACCAA Rev: GACTCTGCT-GCTTCTCCTTG
HSF1	Heat shock factor 1 [*Mus musculus*]	Fwd:CTGAGAAGTGCCT-CAGCGTA Rev: CTCCTGAATGTCCA-GCAGGG

### Statistical analysis

All statistical analyses were performed using the statistical software program GraphPad Prism software (v.6.01; GraphPad Software, Inc., La Jolla, CA, USA). Comparisons among groups were made with one-way ANOVA.

Differences were considered statistically significant at *p < 0.05, **p < 0.01, ***p < 0.001, ****p < 0.0001, *ns* = not significant. Data are given as mean ± Standard Deviation (SD).

### Ethical approval

Our study was conducted in accordance with Animal Research: Reporting of In Vivo Experiments (ARRIVE) guidelines. Interventions and housing of the animals conformed to the Hungarian Laws No. XXVIII/1998 and LXVII/2002 about the protection and welfare of animals, and the directives of the European Union. All animal procedures were approved by the National Scientific Ethical Committee on Animal Experimentation under the No. PE/EA/50-2/2019.

## Results

### Successful transfection of TNBC cell-lines with the HSF1-gene editing lentiviral construct

**Figure 4:**
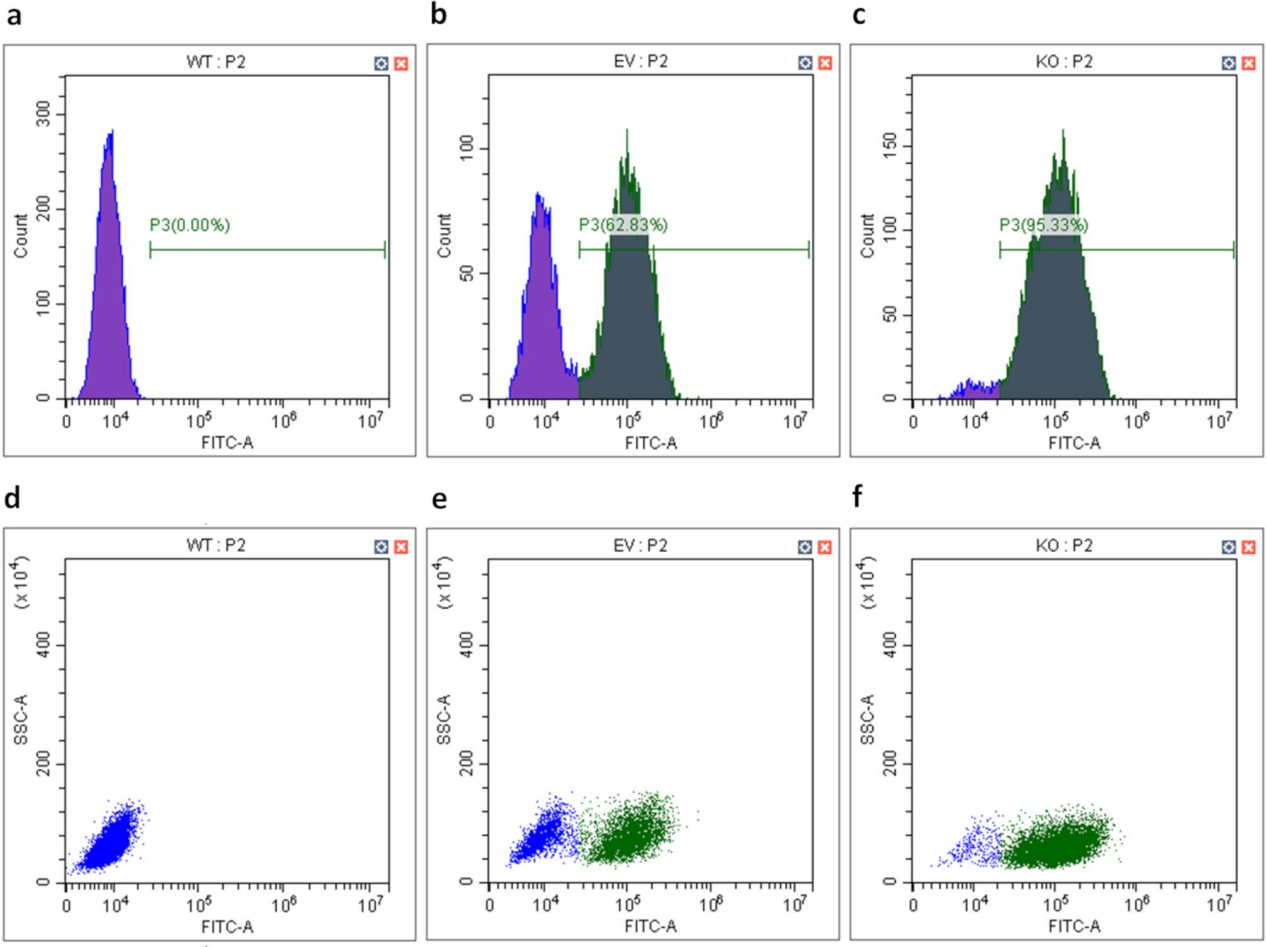
Flow cytometric analysis of transfected 4T1 cells. Flow cytometry histograms (upper row) and dot plots (lower row) (**a**,**d**) wild type (WT), (**b**,**e**) empty vector (EV) and (**c**,**f**) HSF1-KO (KO). The GFP positive cells are indicated with green, and the GFP-negative population is blue. SSC: Side Scatter; P2: second gate; FITC: Fluorescein Isothiocyanate.

The HSF1-knockdown (HSF1-KO) CRISPR/Cas9 construct included a GFP encoding sequence for the selection of transfected cells. As expected, non-transfected wild type (WT) 4T1 cells did not express GFP (Fig. 4a and d). In contrast, more than 95% of 4T1 cells transfected with the active HSF1-KO CRISPR/Cas9 construct successfully expressed GFP, confirming successful transfection (Fig. 4c and f). Transfection with the empty vector (EV) was less effective, resulting in 62.8% of cells being GFP-positive, while 37.2% remained non-transfected (Fig. 4b and e). These non-transfected cells were removed through fluorescent sorting, followed by subsequent propagation in cell culture media supplemented with puromycin.

### Successful reduction of HSF1 and HSP70 expression in transfected TNBC cell-lines

**Figure 5:**
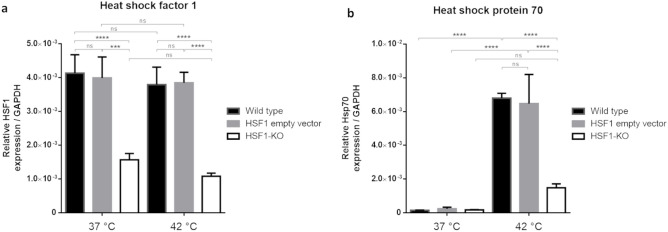
HSF1 and Hsp70 mRNA relative expression at 37 °C and 42 °C in the 4T1 cell line. (**a**) HSF1. (**b**) Hsp70. GAPDH: Glyceraldehyde 3-phosphate dehydrogenase: housekeeping gene. One-way ANOVA, Mean ± SD, n = 3/group, ***p < 0.001, ****p < 0.0001.

HSF1 mRNA levels were comparable in both WT and EV cells, whether maintained at 37 °C or exposed to 42 °C in cell culture (Fig. 5a). Notably, the lentiviral construct led to a substantial downregulation of HSF1 mRNA in the knockdown group (HSF1-KO), as compared to both the wild type and empty vector groups (Fig. 5a). Baseline expression of Hsp70 at 37 °C remained low across all groups (Fig. 5b). Elevating the culture temperature to 42 °C significantly induced Hsp70 upregulation in WT and EV cells. However, this heat-induced response was significantly diminished in the HSF1-KO group (Fig. 5b).

### mEHT-induced tumor growth reduction was enhanced in HSF1-KO tumors

**Figure 6:**
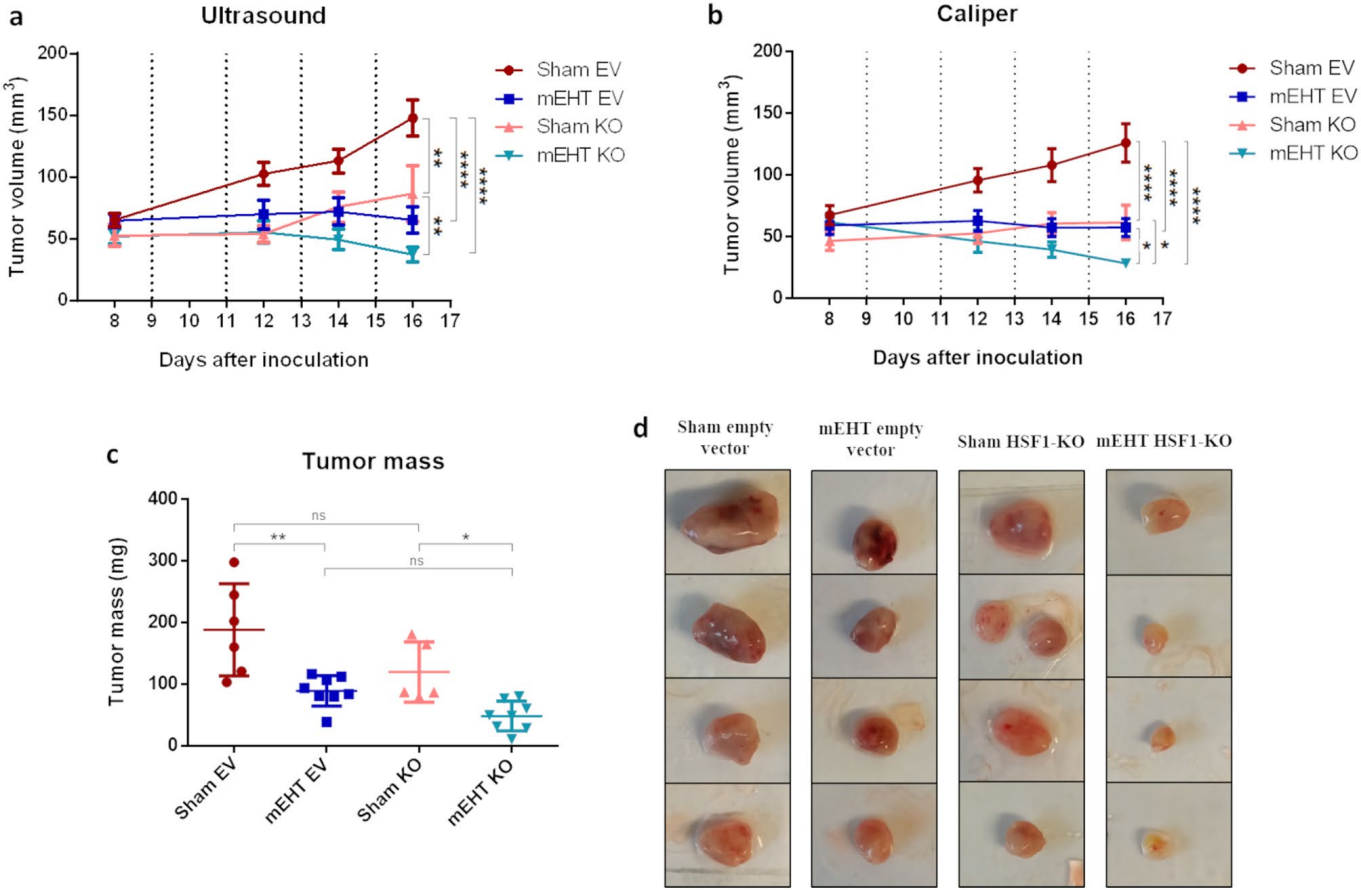
Tumor volume time-course and tumor mass at study termination after 4 mEHT treatments. Tumor growth inhibition as measured by (**a**) ultrasound and (**b**) digital caliper. (**c**) Tumor mass and (**d**) Four representative tumor images of each group (columns) by experiment termination. Mean ± SD, One-Way and TwoWay Anova, Mean ± SD, n = 6–8/group, *p < 0.05, **p < 0.01, ***p < 0.001, ****p < 0.0001.

The Sham EV tumors nearly doubled in volume over the course of the experiment (Fig. 6a and b—red). In contrast, Sham HSF1-KO were smaller and their growth rate was slower than EV tumors (rose). Remarkably, mEHT treated tumors did not grow and their size was reduced after the 4th treatment. The tumor growth rate was significantly slower in the HSF1-KO mEHT-treated group (light blue) compared to mEHT-treated EV group (dark blue). Despite having similar initial tumor volumes, mEHT KO tumors were notably smaller than mEHT EV tumors by the end of the study. Furthermore, supporting the tumor volume data, tumor mass was reduced by both mEHT and HSF-1 KO; yet, the smallest tumors were observed in the mEHT KO group (Fig. 6c and d) by the study termination.

### mEHT-induced tumor destruction was enhanced in HSF1-KO tumors

**Figure 7:**
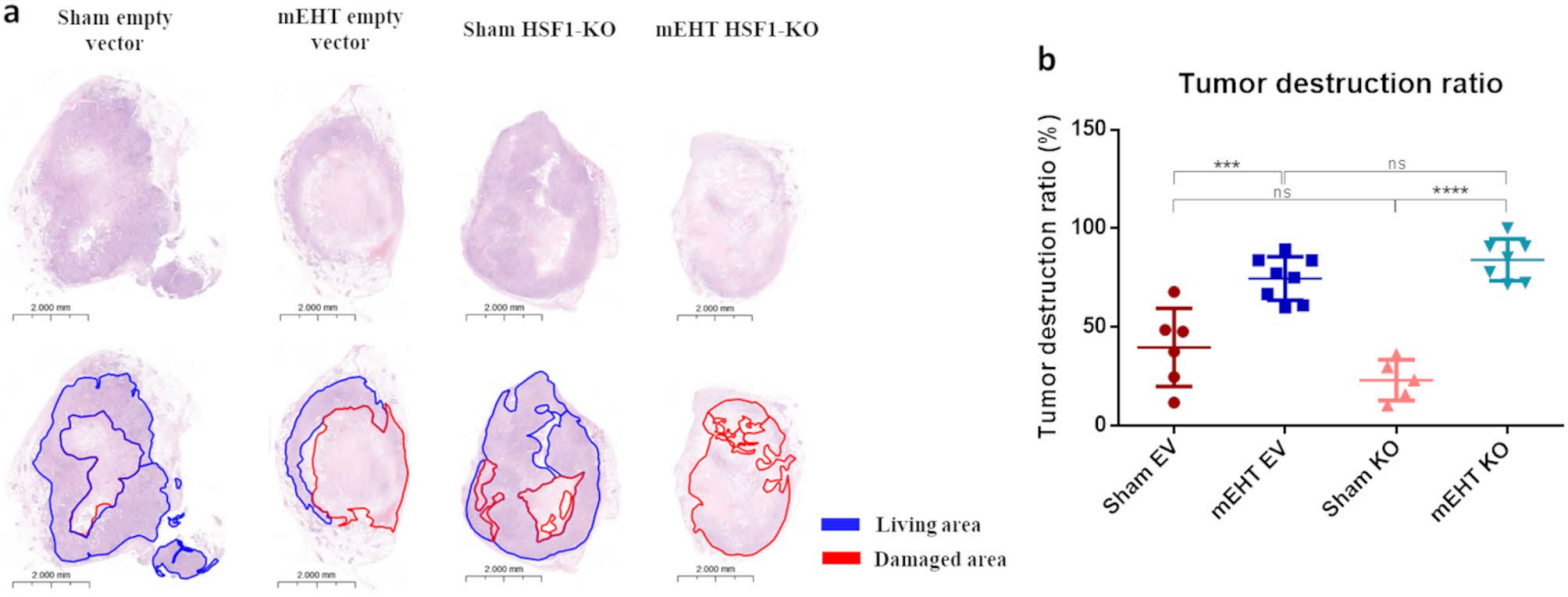
Tumor destruction ratio (TDR) after 4 mEHT treatments. (**a**) Representative tumor images (H&E, 1.0x). (**b**) Quantification of TDR on H&E-stained tumors. One-way ANOVA test, Mean ± SD, n = 5–8/group, ***p < 0.001, ****p < 0.0001.

The area of tumor destruction, quantified as the tumor destruction ratio (TDR), was consistently minimal in all sham tumors, regardless of whether they were EV or KO transfected (Fig. 7a—red marked area). In contrast, mEHT-treated tumors exhibited a substantial increase in tissue damage compared to the sham group (Fig. 7a and b). Notably, there was an increased tendency toward severe tumor damage induced by mEHT in the mEHT-treated HSF1-KO group (mEHT EV: 74.6 ± 11%; mEHT HSF1-KO: 84.1 ± 10.6%, *p* = 0.52, *ns*). It is important to mention that histopathological data is unavailable for one Sham KO and one mEHT KO sample due to their small tumor size.

### HSF1-KO prevented HSF1 and Hsp70 upregulation after mEHT treatment

**Figure 8:**
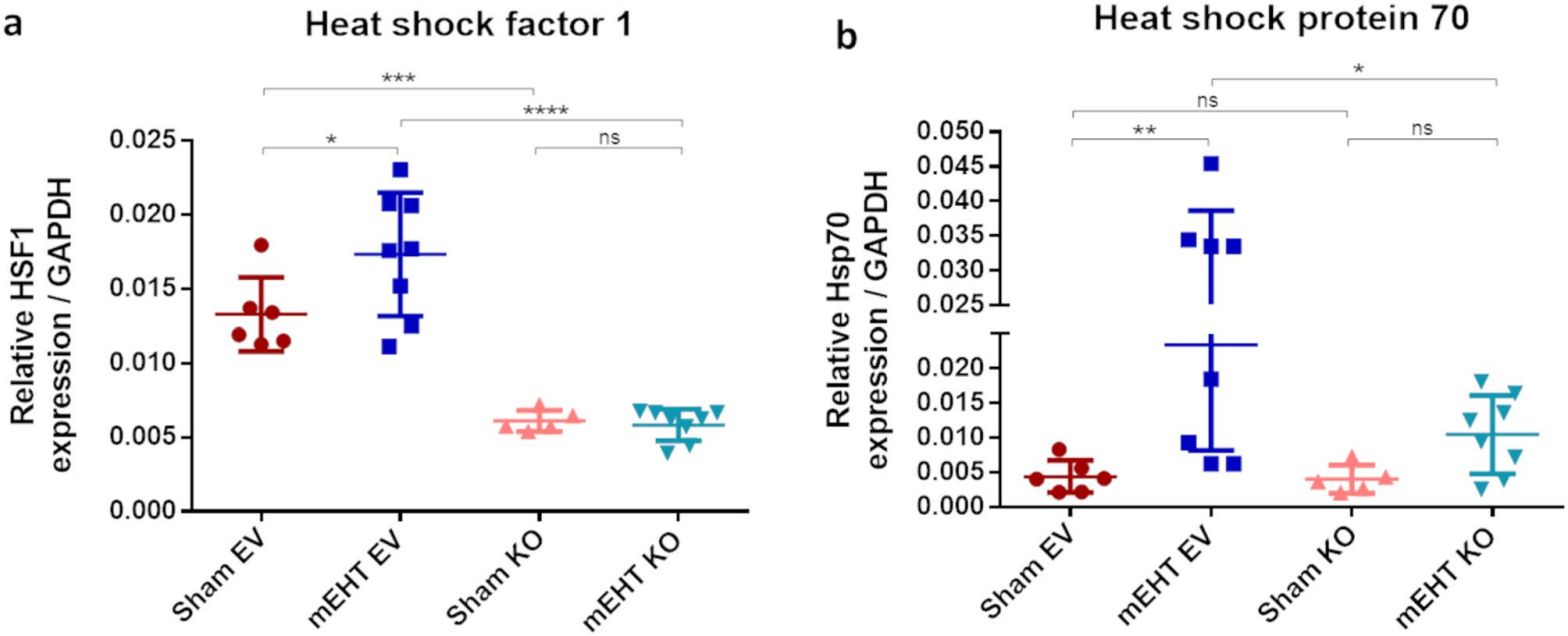
HSF1 and Hsp70 mRNA relative expression after 4 mEHT treatments. (**a**) HSF1. (**b**) Hsp70. GAPDH: Glyceraldehyde 3-phosphate dehydrogenase: housekeeping gene. Oneway ANOVA, Mean ± SD, n = 6–8/group, *ns* = not significant, *p < 0.05, **p < 0.01, ***p < 0.001, ****p < 0.0001.

Relative HSF1 mRNA baseline level, as assessed after four sham or mEHT treatments in EV-treated tumors, was significantly reduced in the KO groups, demonstrating effective silencing (Fig. 8a). HSF1 mRNA was not significantly influenced by mEHT. Contrarily, relative Hsp70 mRNA baseline level, as assessed after four sham treatments, increased significantly in mEHT-treated groups (Fig. 8b). As expected, mEHT stimulated Hsp70 mRNA significantly only in the EV-treated tumors. mEHT did not induce a significant Hsp70 response in HSF1-KO mEHT tumors.

**Figure 9:**
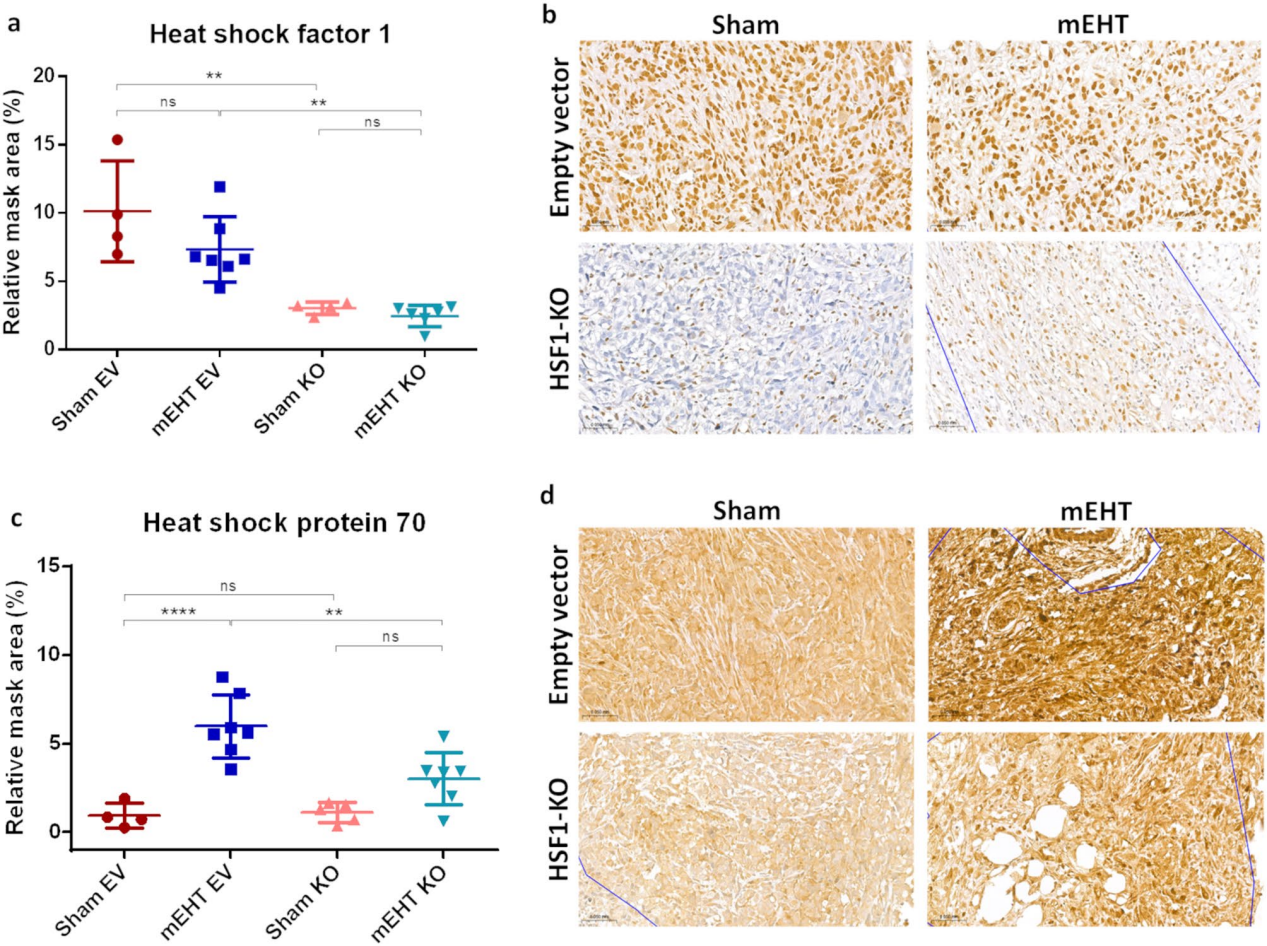
HSF1 and Hsp70 protein detection and quantification with immunohistochemistry after 4 mEHT treatments. (**a**) HSF1 and (**c**) Hsp70 protein quantification. Representative sections of (**b**) HSF1 and (**d**) Hsp70 , 40 × magnification. One-way ANOVA, Mean ± SD, n = 4–7/group, *ns* = not significant, *p < 0.05, **p < 0.01, ***p < 0.001.

Immunohistochemistry (IHC) staining specific for HSF1 was present in most nuclei of empty vector treated tumors (Fig. 9a and b—upper row). Such specific HSF1 staining was not identified in HSF1-KO tumors (Fig. 9b—lower row). HSF1 protein expression was not influenced by mEHT (Fig. 9b—right column).

Hsp70 specific protein staining was intense cytoplasmic staining in mEHT treated tumors. Such specific staining was absent in sham treated tumors (Fig. 9c and d—left column), demonstrating only background staining. Four mEHT treatments induced significant upregulation of Hsp70 specific staining in mEHT treated EV tumors (Fig. 9c and d). Such Hsp70 induction was not significant in the mEHT KO group vs sham-KO demonstrating that HSF1-KO was able to reduce the mEHT induced Hsp70 expression. Hsp70 induction was significantly inhibited in the HSF1-KO *vs* EV mEHT-treated group (Fig. 9c).

### mEHT-tumor growth reduction was synergistically enhanced by the heat shock inhibitor, KRIBB11, after 4 mEHT treatments

**Figure 10:**
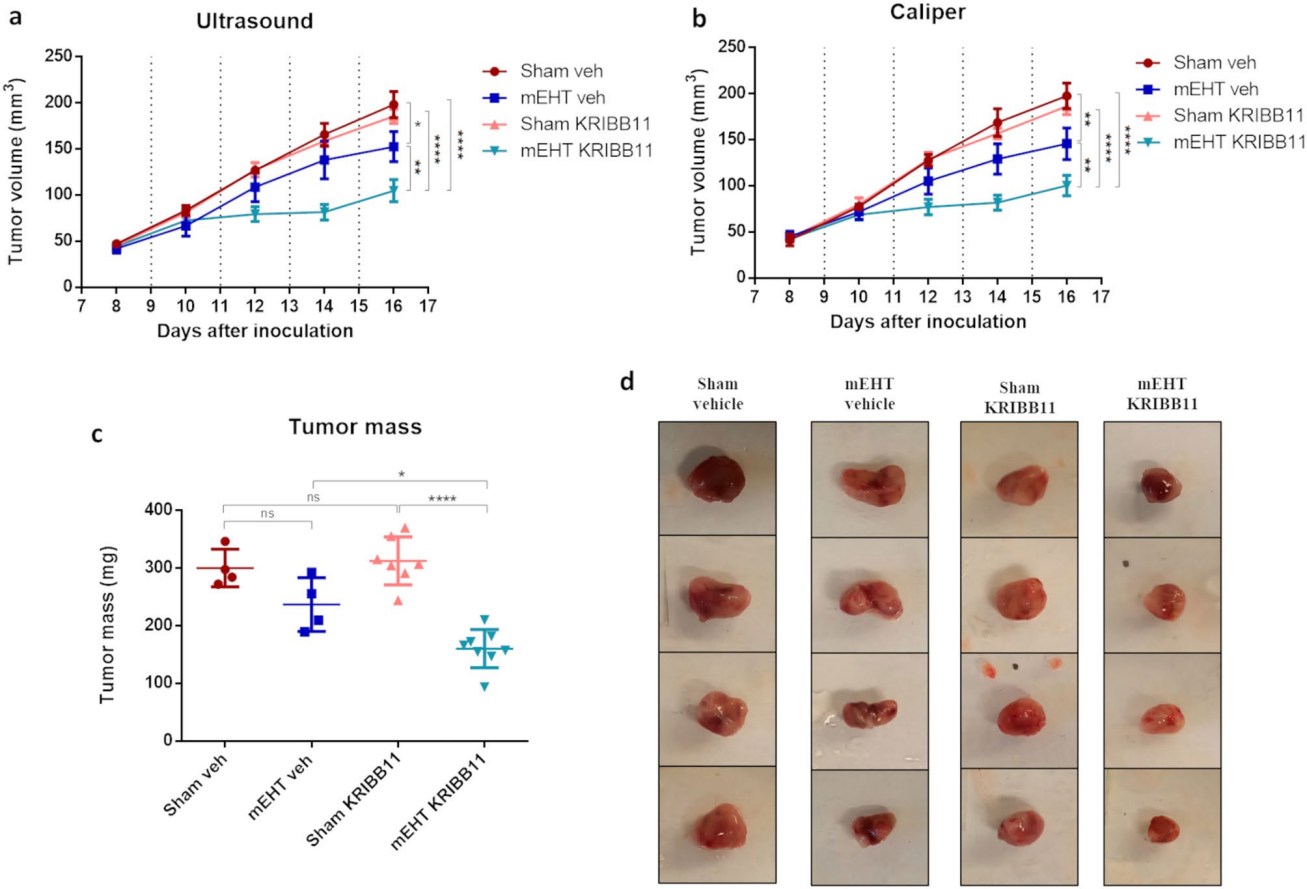
Tumor volume time-course and tumor mass at study termination in the combination therapy experiment. Tumor growth inhibition as measured by (**a**) ultrasound and (**b**) digital caliper. (**c**) Tumor mass and (**d**) Four representative tumor images of each group (columns) by experiment termination. Mean ± SD, One-Way and Two-Way Anova, Mean ± SD, n = 4–8/group, *ns* = not significant, *p < 0.05, **p < 0.01, ****p < 0.0001.

The volume of Sham + Veh tumors increased almost 4-times during the experiment (Fig. 10a and b—red). KRIBB11 monotherapy did not influence tumor growth significantly (rose). However, mEHT treated tumors grew slower (dark blue) and their size was significantly reduced after the 4th treatment (Fig. 10c). Tumor growth rate was further reduced significantly in the KRIBB11 + mEHT co-treated group (light blue). Despite similar tumor volumes at the beginning of the treatments mEHT tumors were significantly smaller than sham tumors at the end of the study. Supporting tumor volume data, tumor mass was reduced only by mEHT but not by KRIBB11 alone. The tumors were significantly the smallest in the KRIBB11 + mEHT co-treated group (Fig. 10c and d) by study termination.

### KRIBB11 prevented Hsp70 upregulation after 4 mEHT treatments

**Figure 11:**
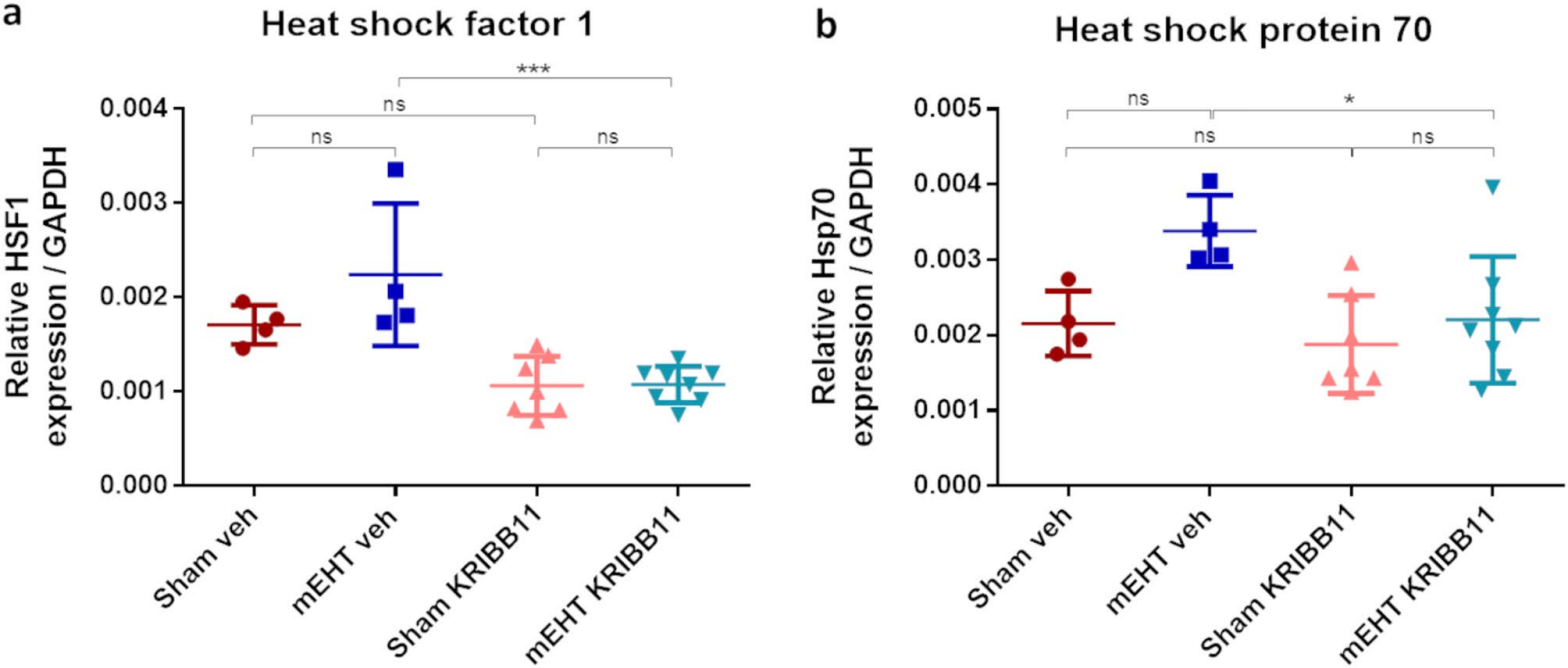
HSF1 and Hsp70 mRNA relative expression after 4 mEHT treatments. (**a**) HSF1. (**b**) Hsp70. GAPDH: Glyceraldehyde 3-phosphate dehydrogenase: housekeeping gene. Oneway ANOVA, Mean ± SD, n = 4–8/group, *ns* = not significant, *p < 0.05, ***p < 0.001.

Relative HSF1 mRNA baseline level as assessed after four sham or mEHT treatments was significantly reduced in the KRIBB11 treated groups demonstrating effective inhibition of the heat shock response in KRIBB11 treated tumors (Fig. 11a). HSF1 mRNA was not influenced significantly by mEHT.

Relative Hsp70 mRNA baseline level was assessed after four sham treatments. Although mEHT induced an increase in Hsp70 mRNA compared to the sham group, this difference did not reach statistical significance (Fig. 11b). In contrast, Hsp70 elevation was absent in KRIBB11 treated tumors receiving mEHT, and Hsp70 mRNA levels were significantly lower in the mEHT + KRIBB11 group compared to the mEHT + Veh group.

**Figure 12:**
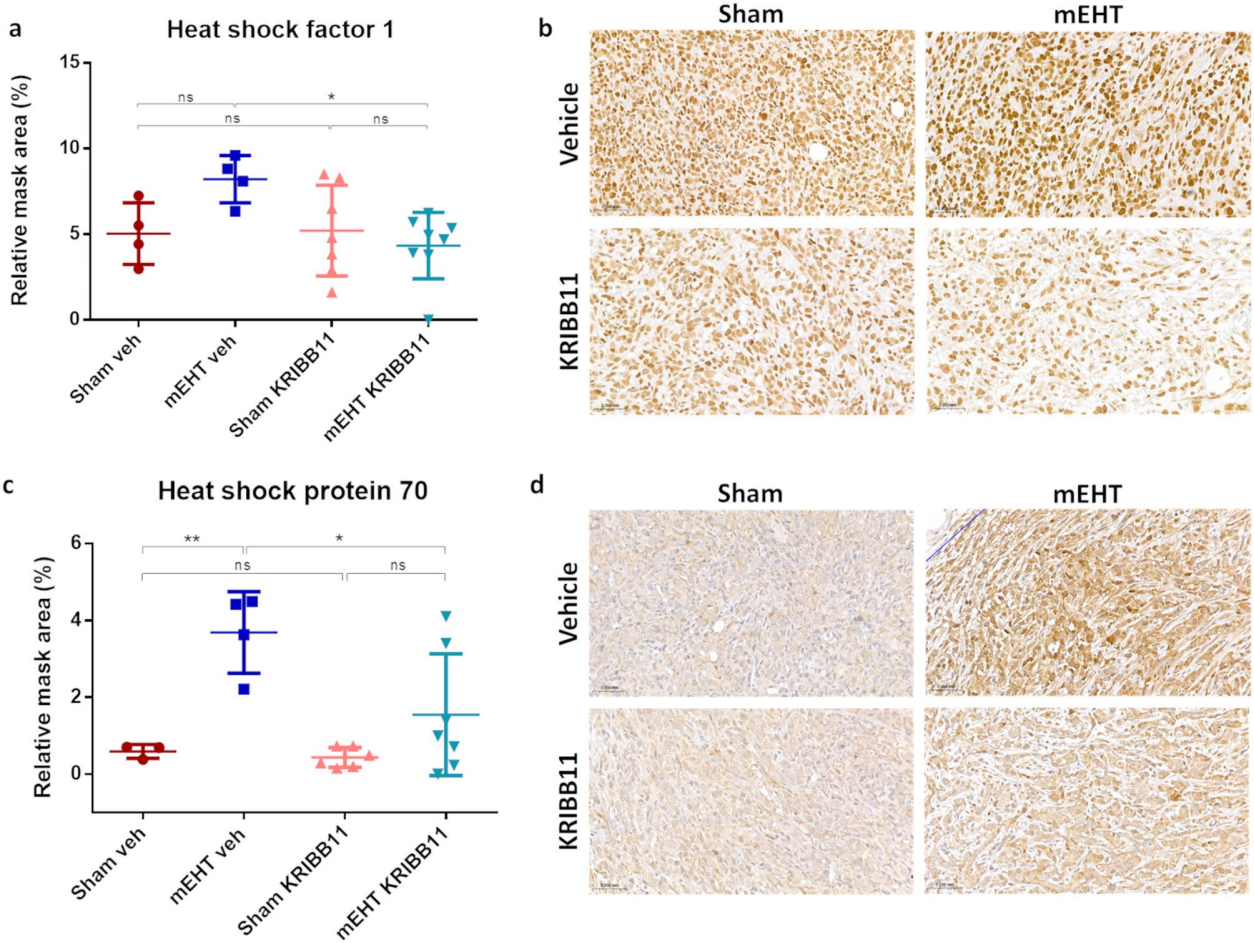
HSF1 and Hsp70 protein detection and quantification with immunohistochemistry after 4 mEHT treatments. (**a**) Heat shock factor 1 (HSF1) and (**c**) Heat shock protein 70 (Hsp70) protein quantification. Representative sections of (**b**) HSF1 and (**d**) Hsp70, 40 × magnification. One-way ANOVA, Mean ± SD, n = 3–8/group, *ns* = not significant, *p < 0.05, **p < 0.01.

In tumors treated with mEHT + Veh, Immunohistochemistry (IHC) staining specific for HSF1 displayed prominent nuclear staining (Fig. 12a and b—upper right panel). In contrast, such specific HSF1 staining was notably less prevalent in KRIBB11 and sham-mEHT treated tumors (Fig. 12b—lower panel). Remarkably, mEHT treatment did not significantly alter HSF1 protein expression levels (Fig. 12b).

Intense cytoplasmic staining specific to Hsp70 protein was observed exclusively in tumors treated with mEHT monotherapy (Fig. 12c and d—right columns). In contrast, such distinctive staining was notably absent in sham-treated tumors, indicating only background levels of Hsp70 (Fig. 12c and d—left columns). The mEHT-induced upregulation of Hsp70 was significantly attenuated by co-treatment with KRIBB11, emphasizing the inhibitory effect of HSF1 on mEHT-induced Hsp70 expression (Fig. 12c).

### Slightly increased tumor destruction tendency was observed in tumors treated with combined therapy

**Figure 13:**
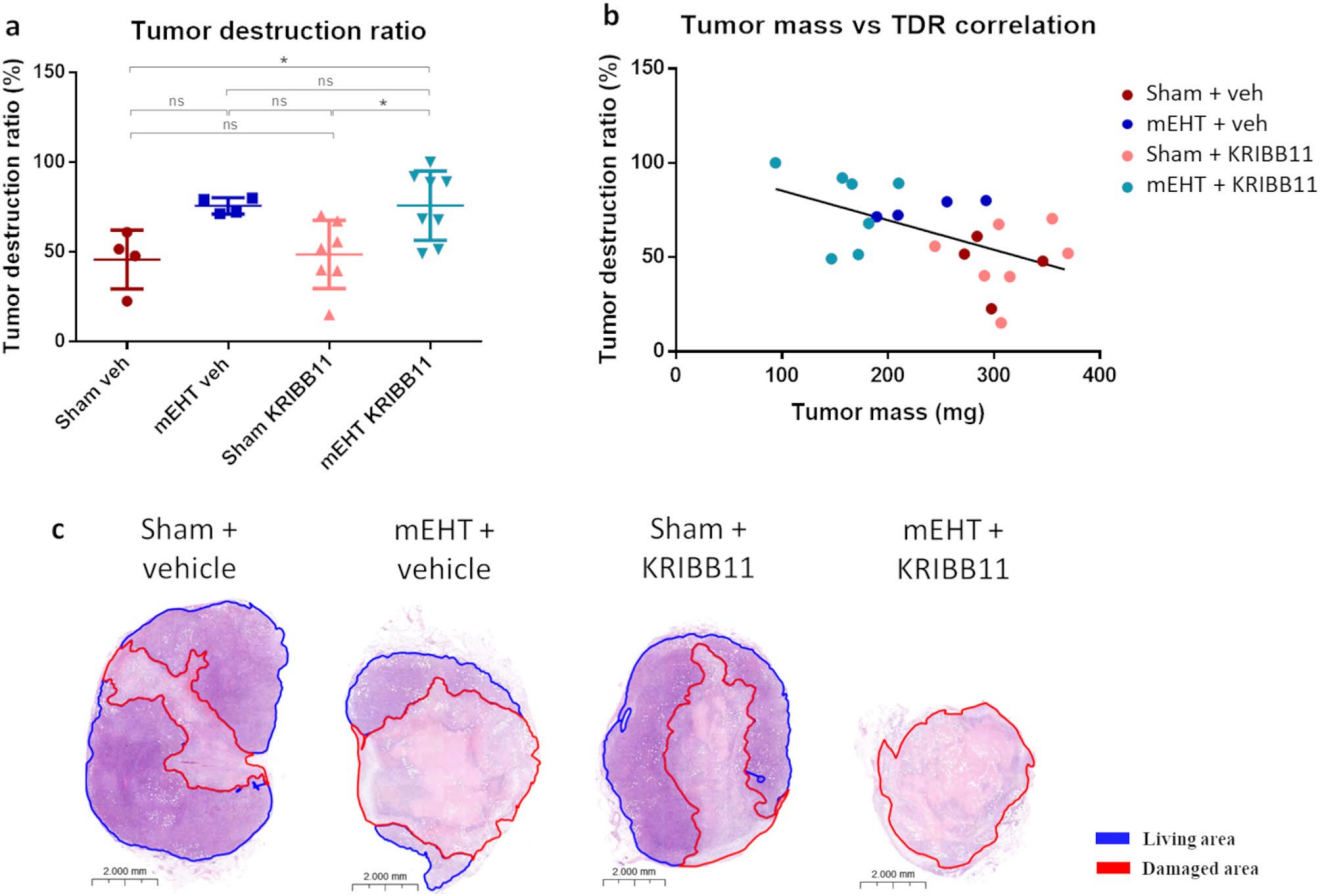
Tumor destruction ratio (TDR) after 4 mEHT treatments. (**a**) Quantification of TDR on H&E-stained tumors. (**b**) Scatter diagram and linear regression line showing negative correlation between tumor destruction ratio (TDR: Y axis) and tumor mass (X axis). (**c**) Representative tumor images (H&E, 1.0x). One-way ANOVA test, Mean ± SD, n = 4–8/group, *ns* = not significant, *p < 0.05.

The Tumor Destruction Ratio (TDR) was relatively small in all sham tumors (Fig. 13c—red marked area). Notably, there was significant tissue damage observed in mEHT-treated tumors (Fig. 13a and c). While a subset of mEHT + KRIBB11 treated tumors demonstrated enhanced damage, another subset exhibited a smaller TDR, resulting in a non-statistically significant overall effect. It is worth noting that the core damaged area in the tumors exhibited a negative correlation with tumor mass, with larger tumors demonstrating a higher TDR (Fig. 13b). The most pronounced tumor destruction relative to tumor size was observed in the mEHT + KRIBB11 combination therapy group, highlighted by light blue dots.

## Discussion

Modulated eletcro-hyperthermia (mEHT) is a loco-regional non-invasive cancer therapy which has been successfully applied in vitro, in vivo, and in the clinics for over 20 years^15^. The mEHT treatment triggers apoptosis and necrosis of cancer cells via non-thermal effects such as membrane perturbations (similarly to electrophoresis), and the radiofrequency field also induces temperature increase to 42 °C^16^. The high energy absorbed by cancer cells and specifically cancer cell membranes lipid rafts consequently disrupts membrane arrangement and integrity on the basis of its elevated oxidative glycolysis (Warburg effect), ion concentration, and conductivity compared to adjacent normal tissues^36^ and inducing anti-cancer responses^12^. However, it is known that mEHT provokes cell- and heat-stress activating the heat shock response^11^. In our previous studies, we demonstrated that mEHT induced the heat shock response in our mouse cancer model, resulting in strong upregulation of Hsp70^17,23^. Indeed, massive induction of Hsp70 has been reported in other cancer models treated with mEHT such as colorectal^37^, and melanoma^38^ models. It is worth noting that Hsp70, a crucial molecular chaperone, typically maintains low or undetectable levels in unstressed cells^39^. The transcription of Hsp70 is triggered by the nuclear translocation of heat shock factor 1 (HSF1), a master regulator of the heat shock response^40^. When activated, HSF1 binds to the heat shock response elements (HSEs) within promoters of several Hsps and initiates Hsp transcription^41^. It has been demonstrated in a wide range of cancer types that HSF1 has a cytoprotective activity and supports cancer cell proliferation, survival, invasion, and metastasis^42^. Targeting HSF1 in cancer therapy has been suggested before^27^, and in HSF1-knockdown experiments, mammary^43^, liver^44^, and skin^32^ carcinogenesis was inhibited. We therefore hypothesized that the inhibition of mEHT-induced heat shock response by inhibiting HSF1 would enhance the therapeutic potential of mEHT. As a proof-of-concept, we used genome editing tools to knockdown HSF1. To investigate the translational potential, we also studied a small molecule inhibitor (KRIBB11), aiming to inhibit HSF1 and thus enhance the anticancer effects of mEHT treatment.

The integration of mEHT with conventional cancer treatment modalities presents a promising avenue in the quest for enhanced therapeutic efficacy. mEHT, known for its ability to selectively elevate temperatures in tumor tissues, can act synergistically and complementarily to radiotherapy and chemotherapy. A slight temperature elevation is expected to enhance blood flow, facilitating improved oxygenation in the affected area. This, in turn, can augment the efficacy of gold-standard cancer treatments^45^. The synergistic interplay of ionizing, thermal, and non-thermal effects promotes immunogenic cell death in malignant cells. This is achieved by exciting lipid rafts through mEHT-induced heating and inducing double-strand DNA breaks with radiotherapy^46^. Furthermore, studies have demonstrated the additional benefits of mEHT in conjunction with conventional therapies, leading to increased treatment response, improved overall survival, and a lack of side effects^47–49^. Thereofre, this combinatorial strategy holds the promise of not only improving the local control of tumors but also potentially minimizing side effects.

In the present study, CRISPR/Cas9-mediated HSF1 knockdown was successful in 4T1 murine breast cancer cells. While the use of CRISPR to completely ablate genes can be challenging, as this system rarely eliminates the expression of a target gene entirely, leading to low knockdown efficiencies (< 80%)^50–54^, we achieved an impressive knockdown efficacy of over 95% (Fig. 4). This high efficacy was evidenced by the GFP-positivity of transduced cells after antibiotic selection (puromycin) and FACS sorting. However, these strategies seemed to be less effective in the empty vector group (62.83%). This group used a non-targeting guide RNA with no specific target site on the entire genome, designed to serve as a control group with a wild type phenotype. The genome editing using the CRISPR/Cas9 lentiviral empty vector did not enable gene truncation via polymerase chain reaction (PCR) (Fig. 5). On the other hand, we achieved a very strong HSF1 knockdown efficiency as demonstrated by flow cytometry and confirmed by real-time PCR (Fig. 8). Immunohistochemistry analysis of in vivo tumors further corroborated the HSF1 knockdown (Fig. 9a and b). Baseline HSF1 expression was reduced and hyperthermia-induced Hsp70 upregulation was inhibited in HSF1-KO cells.

mEHT cancer selectivity and its inhibition of tumor growth have been reported in a wide range of cancer types^55^. Previously we also demonstrated inhibition of tumor growth by mEHT monotherapy in our murine breast cancer model^17,23^. In this study, we observed a significant enhancement in tumor growth inhibition with mEHT treatment in HSF1-KO cells. HSF1-KO tumors exhibited a size reduction of over 50% compared to non-mEHT-treated empty vector tumors (Fig. 4). This aligns with previous findings suggesting the essential role of the HSF1 gene in cancer development, as HSF1-KO tumors naturally grow at a slower rate than empty vector tumors^33^. Despite this fact, mEHT further amplified the reduction in tumor growth in the KO group. Therefore, the importance of HSF1 supporting carcinogenesis can be demonstrated by the susceptibility reduction of HSF1-KO cells to cancer formation^56^.

Based on our previous data^17^ and the HSF1 knockdown study, we hypothesized that the inhibition of heat shock response (HSR) by KRIBB11, a specific inhibitor of HSF1^57^, could potentiate the anticancer effects of mEHTin tumor allografts. Mouse cancer xenograft studies have demonstrated reduction of cancer growth in samples treated daily with KRIBB11 dose in human colon cancer^28^, breast cancer^58,59^, human myeloma^60^, bladder cancer^61^, and hepatocellular carcinoma^62^, both alone or in combination with other chemical compounds. When in combination, KRIBB11 efficacy was increased^58^. Indeed, we propose that KRIBB11 has translational potential when applied in combination with mEHT treatments, as the combined therapy demonstrated synergism in tumor growth inhibition (Fig. 10). Here we established the synergism between four-mEHT treatments and daily dose of KRIBB11 at 50 mg/kg for 8 days, as demonstrated by tumor volume and tumor mass reduction in the combined therapy group. Using the average of tumor masses for each treatment group (Sham + Vehicle: 300.0 mg, mEHT + Vehicle: 237.0 mg, Sham + KRIBB11: 312.3 mg, and mEHT + KRIBB11: 160.3 mg), we calaculated the expected combined effect based on individual mEHT and drug effects based on the Bliss Independence model^63,64^. Our analysis revealed that the observed effect of mEHT + KRIBBB11 (*E*_*mEHT*+*KRIBB11*_ = 0.466) exceeded the expected combined effect (*E*_*Bliss*_ = 0.243). Specifically, *E*_*mEHT*+*KRIBB11*_ > *E*_*Bliss*_, indicating a potential synergistic interaction between mEHT and KRIBB11. This suggests that the combined treatment may exert a more pronounced inhibitory effect on tumor growth than antecipated based on the individual effects of each agent alone.

However, KRIBB11 alone was not able to reduce cancer growth in monotherapy (Fig. 10c). Carpenter et al*.* reported similar findings, stating that KRIBB11 did not significantly inhibit tumor growth in a breast cancer model unless combined with an AKT inhibitor^58^. In another study a dose of 50 mg/kg of KRIBB11 did not reduce myeloma xenograft growth, whereas a dose of 65 mg/kg proved effective^60^.

The effects of KRIBB11 abovementioned were achieved by following the protocol established by Yoon et al*.* which involved daily administration of KRIBB11 over a period of 18 days^28^. Due to our experiment’s shorter duration (8 days), our mice received fewer KRIBB11 injections. This decision was based on our previous findings, which demonstrated that prolonged mEHT treatments could lead to substantial damage in tumor tissue. This damage hinders the isolation and detection of RNA from treated tumors, thereby impeding molecular analysis^17^. This may also account for the observed lack of significant tumor growth inhibition with KRIBB11 monotherapy.

While previous studies have demonstrated that mEHT induces the heat shock response mainly by upregulation of Hsps^11,12^, we observed that mEHT may not directly lead to significant changes in HSF1 mRNA (Fig. 11a) and protein levels (Fig. 12a). Instead, the regulation of HSF1 might primarily occur through its cellular localization, particularly its movement from the cytoplasm to the nucleus in response to stress^65^. This nuclear translocation of HSF1 is a crucial step in initiating the transcription of additional Hsps.

Corroborating with the HSF1-KO TDR data (Fig. 5), the damaged area observed in the combined mEHT and KRIBB11 therapy did not demonstrate statistically significant differences compared to mEHT + vehicle (Fig. 13). While tumor volume and mass reduction demonstrated synergistic enhancement of the mEHT effect with additional KRIBB11 therapy, the TDR results were not as pronounced. This could be attributed to the extensive destruction observed in Sham tumors, leading to spontaneous necrosis due to increased tumor size, as previously noted by our group^17^. The elevated mitochondrial metabolism of 4T1 cancer cells might contribute to necrosis development as a result of low oxygen levels and nutrient supply^66^. Therefore, the damage magnitude may be related to tumor size. Indeed, our study revealed that larger tumors tended to have moderate TDR’s (Fig. 13b). This trend was primarily due to their large size, with most of them belonging to the Sham groups (vehicle and KRIBB11). Consequently, the necrosis observed at the core of Sham tumors was a direct result of cancer outgrowth (Fig. 13b, red and pink dots). In contrast, mEHT-treated tumors exhibited a notably high TDR along with reduced mass (Fig. 13b, dark blue and light blue dots). From this, we infer that necrosis observed in mEHT groups (both vehicle and KRIBB11) arose from the cancer-killing effect of mEHT. Furthermore, this effect was amplified by administration of KRIBB11 (Fig. 13b, light blue dots). This suggests that the increase in cancer core destruction in Sham groups is primarily linked to tumor size, whereas in mEHT groups, it is more likely due to the treatment itself.

In conclusion, we have demonstrated that the combination of modulated electro-hyperthermia (mEHT) with the CRISPR/Cas9 gene-editing technique significantly enhances its anticancer effects in vivo. Specifically, the knockdown of heat shock transcription factor 1 (HSF1) led to the inhibition of tumor growth and a reduction in Hsp70 upregulation induced by mEHT. Moreover, the administration of the specific heat shock inhibitor, KRIBB11, further amplified the therapeutic impact of mEHT. These findings suggest a potential synergy between KRIBB11 and established clinical anticancer therapies like mEHT. Consequently, KRIBB11 holds promise for translation into clinical applications, offering a potentially impactful addition to cancer treatment modalities.

### Supplementary Information

Supplementary Figures.

## Data Availability

All data associated with this study are presented in the paper. The data that support the findings of this study are available from the corresponding author upon reasonable request.

## Abbreviations

ER: Estrogen receptor
EV: Empty vector
HER2: Human epidermal growth factor receptor 2
HSF1: Heat shock factor 1
Hsp: Heat shock protein
HSR: Heat shock response
IHC: Immunohistochemistry
KO: Knockdown
mEHT: Modulated electro-hypertermia
PR: Progesterone receptor
RF: Radiofrequency
shRNA: Short hairpin RNA
TDR: Tumor destruction ratio
TNBC: Triple-negative breast cancer
Veh: Vehicle
WT: Wild-type
