# Supplementary material for: Heat shock factor 1 inhibition enhances the effects of modulated electro hyperthermia in a triple negative breast cancer mouse model
Source: Sci Rep. 2024 Apr 8;14:8241. doi: 10.1038/s41598-024-57659-x (PMC11002009; doi:10.1038/s41598-024-57659-x)
Supplement: Supplementary file 1 — Supplementary Figures. [file 41598_2024_57659_MOESM1_ESM.docx]

Heat Shock Factor 1 Inhibition Enhances the Effects of Modulated Electro Hyperthermia in a Triple Negative Breast Cancer Mouse Model

Pedro H. L. Viana^a^, Csaba A. Schvarcz^a,b^, Lea O. Danics^a^, Balázs Besztercei^a^, Kenan Aloss^a^, Syeda M. Z. Bokhari ^a^, Nino Giunashvili^a^, Dániel Bócsi^a^, Zoltán Koós^a^, Zoltán Benyó^a,b^, Péter Hamar^a^

*^a^Institute of Translational Medicine, Semmelweis University, Tűzoltó utca 37-47., Budapest, 1094, Hungary ^b^* *HUN-REN-SU Cerebrovascular and Neurocognitive Diseases Research Group, Budapest, Hungary, Tűzoltó utca 37-47., Budapest, 1094, Hungary*

**Supplementary figures**


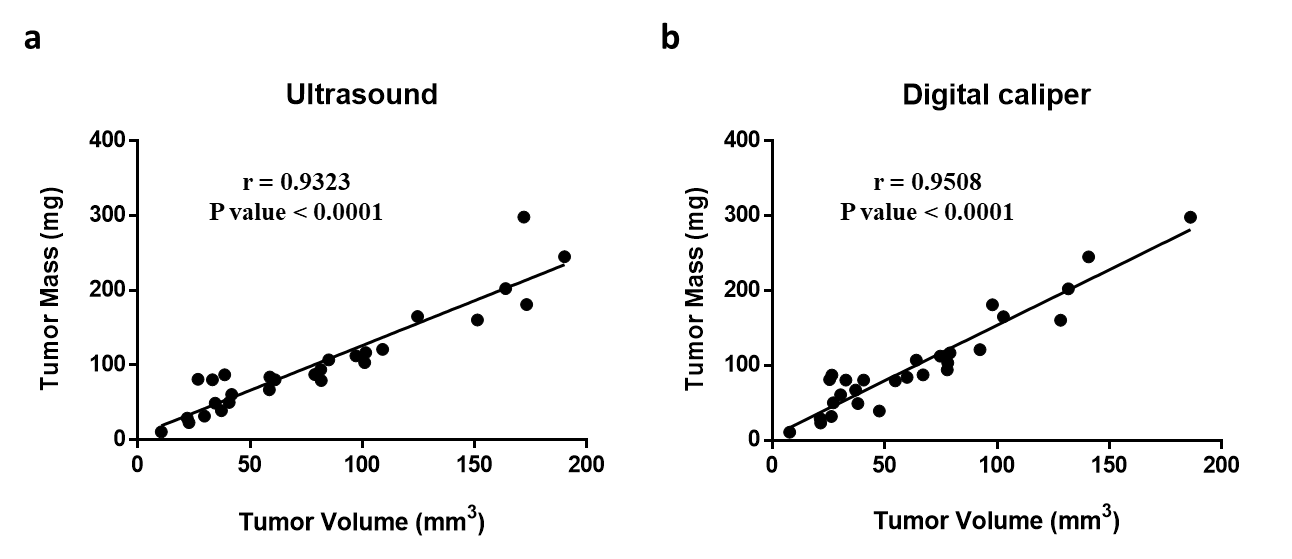


**S1.** Correlation between tumor mass and tumor volume by **a** ultrasound and **b** digital caliper for mEHT + HSF1-KO experiment.


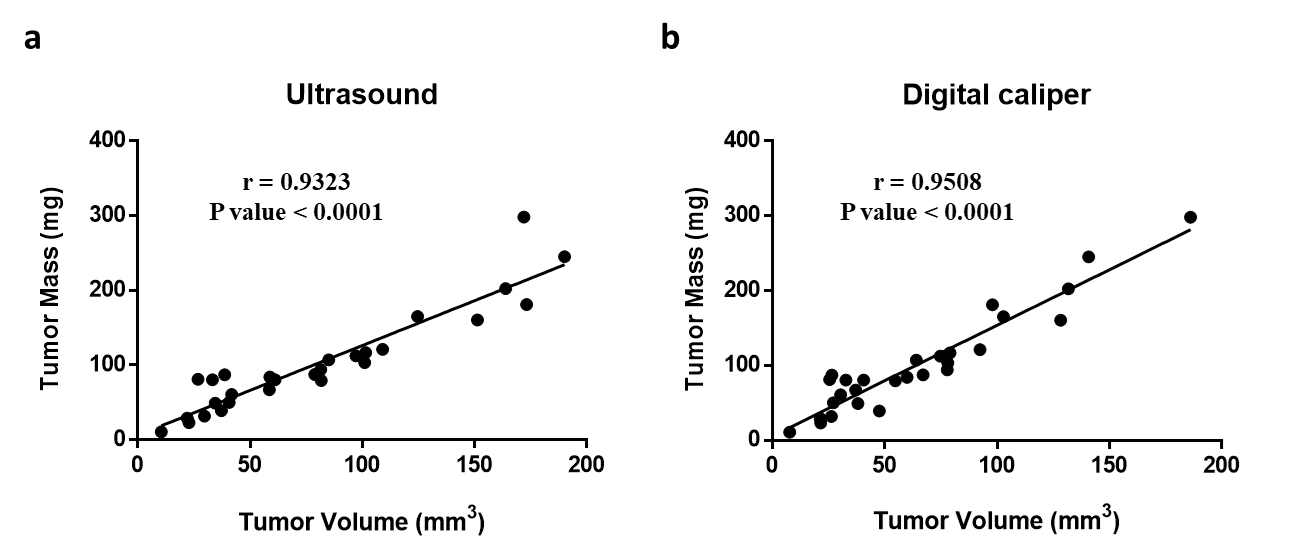


**S2.** Correlation between tumor mass and tumor volume by **a** ultrasound and **b** digital caliper for mEHT + KRIBB11 experiment.
